# Fetus in Fetu in an Adult Female and Brief Review of Literature

**DOI:** 10.1155/2021/6660277

**Published:** 2021-02-11

**Authors:** Saroj Sharma, Prashant Kumar Gupta, Basanta Regmi, Aarti Gupta, Upasana Lamichhane

**Affiliations:** ^1^Department of Radiology, National Academy of Medical Sciences, Bir Hospital, Mahaboudha, Kathmandu, Nepal; ^2^KIST Medical College and Teaching Hospital, Gwarko, Lalitpur, Nepal; ^3^Tulsi Hospital Pvt. Ltd., Tulsipur, Dang, Lumbini Province, Nepal

## Abstract

Fetus in fetu (FIF) is a very rare condition in which malformed fetus is found within the body of a living twin, most commonly within the retroperitoneum. It is a parasitic fetal twin of a diamniotic, monozygotic type. It should be differentiated from teratoma by the presence of organized vertebral column and appropriately arranged other organs or limbs around it. There is no such axial arrangement in teratoma, which has also got definite malignant potential. We report a case of FIF in a 21-year-old lady who presented late with nonspecific abdominal symptoms. Preoperative diagnosis of FIF in this case was made on computed tomography, and the patient underwent exploratory laparotomy with complete excision of mass. The excised mass in a sac was proven to be FIF on the basis of gross and histopathological examination. Surgical excision is the ideal treatment even teratoma being the differential diagnosis.

## 1. Introduction

Fetus in fetu (FIF) is an extremely rare developmental condition. It occurs in about 1 in 500 000 live births, and around 200 cases have been reported in medical literature to the best of our knowledge. FIF is usually retroperitoneal, but it has been reported at various sites right from the cranial cavity to the scrotal sac. It is usually surrounded by a membrane analogous to amniotic sac [1–3]. There is absence of independent circulatory system which explains the subsequent growth retardation. Developmentally, it has gone through the stage of primitive streak and that is why it has vertebral body and organs arranged around the axis. This differentiates it from fetiform teratoma which has no such organization. The embryo pathogenesis of FIF has been described by the “included-twin” theory, which explains FIF as a diamniotic, monochorionic, monozygotic twin embodying into the body of the host twin after colligation of the vitelline circulation [4]. It was first described by Johann Friedrich Meckel in the late 18th century [5]. Majority of cases have been described in neonates and children, and only few cases have been reported after the age of 15 years and most of them are male [4].

Reviewing the literature, we aim to report this case since this case is one of the only few cases of FIF in a female gender of early twenties.

## 2. Case Presentation

A 21-year-old woman presented to the surgery outpatient department (OPD) with clinical history of right upper abdominal lump for 6-7 years, which was gradually increasing in size. It was associated with dull aching type of pain, which was on and off in nature. There was no history of fever, weight loss, altered bowel habits, or urinary symptoms. Her menstrual history was within the normal limits. There was no history of twin birth or teratoma in the family. Her general physical examinations were within the normal limits. On abdominal examination, as done in surgery OPD, there was a lump involving the right upper quadrant of the abdomen. It had ill-defined margins with soft to firm consistency and irregular surface. It was not moving with respiration. On evaluation, beta-human chorionic gonadotropin (*β*-HCG), alpha fetoprotein (AFP), and routine blood investigations were found within the normal limits. For further diagnostic evaluation, ultrasonography (USG) was done. Prior imaging examinations were not done in this patient. USG showed a large, ill-defined heterogeneous mass in the right side of the abdominal cavity adjacent to the liver, having multiple hypoechoic areas and echogenic structures with no internal vascularity. With inconclusive diagnosis, she was admitted in surgery ward for further evaluation.

**Figure 1:**
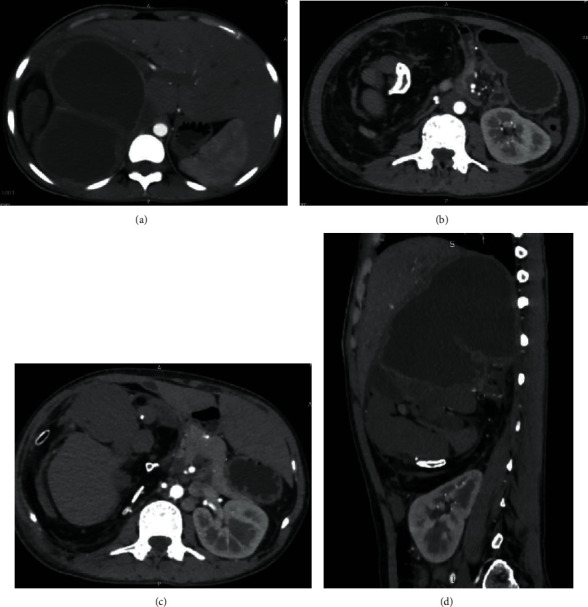
Abdominal computed tomography shows a large heterogeneous retroperitoneal mass with cystic, calcified, soft tissue and lipomatous components (a). Large cystic component (b). Well-formed vertebral body with surrounding lipomatous tissue (c). Well-formed ribs and long bone (d). Inferior displacement of the right kidney by mass.

**Figure 2:**
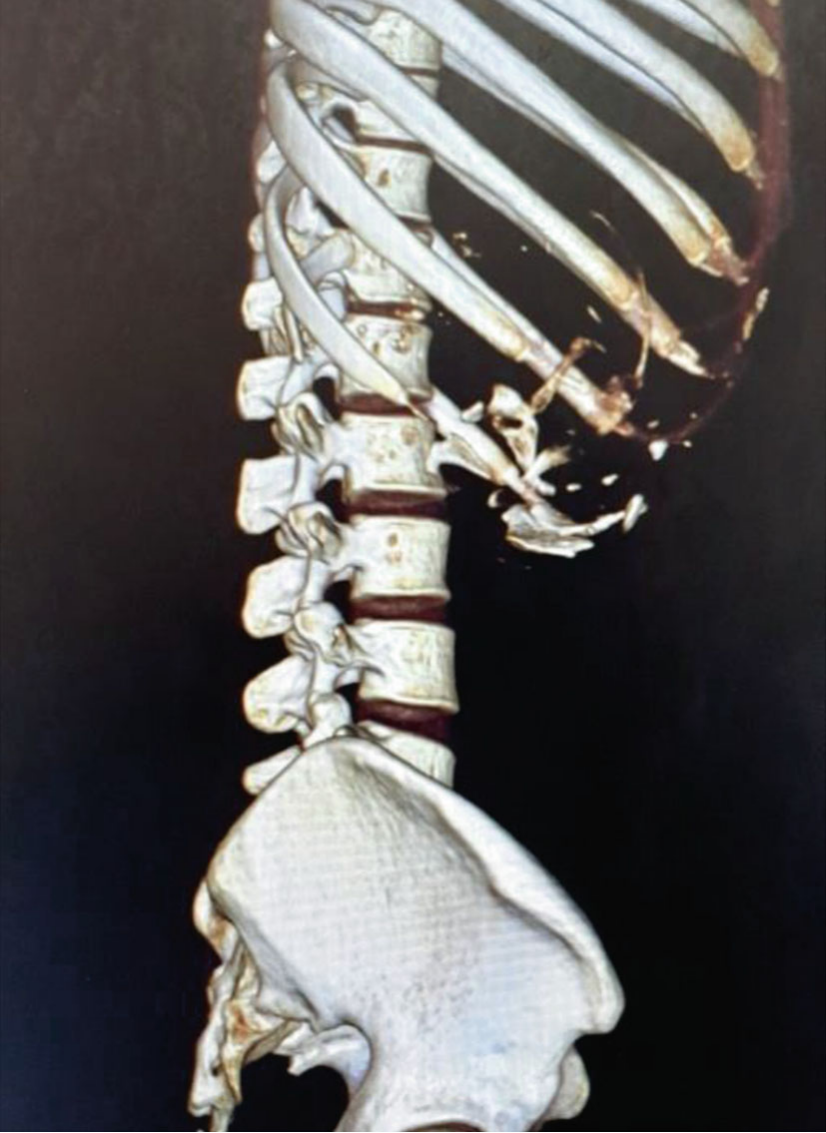
Three-dimensional reconstruction CT image showing bony outlines of fetus in fetu.

Later, contrast enhanced CT scan of the abdomen and pelvis revealed a large heterogeneous retroperitoneal mass in the right side of the abdomen, measuring approximately 22.5 × 14.1 × 12.5 cm having cystic areas, fat attenuating areas, foci of calcifications, malformed skeletal parts resembling long bones, ribs and vertebrae, and heterogeneous soft tissue fetal components. Soft tissue components showed minimal peripheral enhancement. The mass was lying predominantly in the posteroinferior aspect of the right lobe of the liver causing its displacement anteriorly and to the left side and extending superiorly up to D8 vertebral body causing superior displacement of the right hemidiaphragm. It has had extension inferiorly up to L3 vertebral body with inferiorly displaced and malrotated right kidney. Inferior vena cava was displaced anteriorly and medially. Bowel loops were also displaced inferiorly and to the left side. It has had maintained fat plane with the surrounding host organs (Figure 1). The uterus and bilateral adnexa were normal. With the features of well-organized axial skeleton and long bones (Figure 2), CT findings were suggestive of fetus in fetu with various mass effects. Organized teratoma was kept as differential diagnosis.

**Figure 3:**
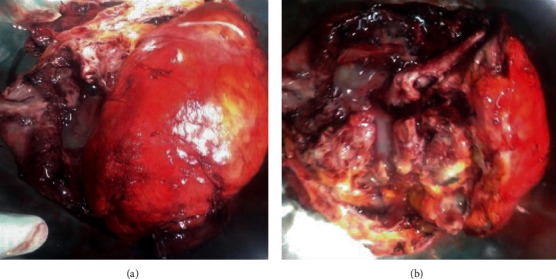
(a, b) Gross postoperative specimens showing heterogeneous soft tissue mass containing fatty areas and bony and cartilaginous tissues.

Mass being huge with its retroperitoneal location, difficult surgical planes, and surrounding structural relationship, laparotomy was planned over laparoscopy for further diagnostic and therapeutic purposes. It was performed and mass was excised completely. Grossly, it was enclosed in a sac containing fat, malformed bony and cartilaginous tissues, soft tissue components along with large amount of serous fluid (Figure 3). The uterus and bilateral adnexa were grossly unremarkable. Total operative time was around 4 hours, and total amount of blood loss was approximately 300 ml. Her total postoperative hospital stay was 5 days and uneventful.

**Figure 4:**
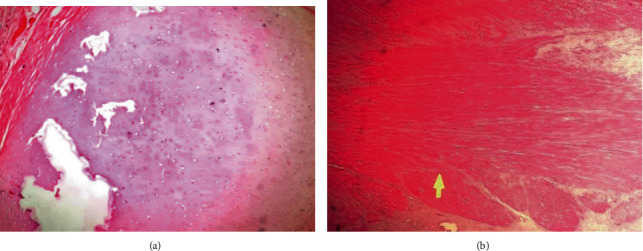
(a) Microscopic section showing well-formed cartilaginous tissue (H and E, ×100). (b) Microscopic section showing well-formed skeletal muscle tissues (H and E, ×100).

The mass was sent for histopathological examination. Histopathology showed mixture of variable tissues in variable proportion. There were areas of tissues lined by respiratory epithelium, intestinal epithelium, and stratified squamous epithelium. Muscle bundles, cartilages, bones, glial tissues with psammoma bodies, adipocytes, thyroid follicles, and lymphoid aggregates were also seen. Multiple cystic spaces lined with ciliated columnar epithelium were also noted. However, there were no immature components and no features of malignancy seen (Figure 4). Gross and histopathological findings of mass confirmed it to be fetus in fetu.

She has been under regular follow-up with normal level of tumor markers *β*-HCG and AFP, and she is doing well. Her last follow-up was around 3 months after the surgery.

## 3. Discussion

Fetus in fetu occurs secondary to abnormal embryogenesis in a monochorionic diamniotic pregnancy in which a malformed parasitic twin is found inside the body of its partner as an abdominal fetiform mass [1–3]. FIF presents in various age groups with predominance in infancy, and in the majority of cases, it is diagnosed in patients younger than 18 months of age, with very few cases reported in adults. To the best of our knowledge and review, there are no more than 10 cases reported on the adult population [3–5]. This is one of the adult females to have been detected with a fetus in fetu. It commonly presents as an asymptomatic abdominal mass. In our case, the patient presented with lump in the right upper quadrant. The most common location of FIF is the retroperitoneum. However, other sites like the mediastinum, neck, sacrococcygeal region, back, scrotal sac, cranial cavity, and oral cavity have also been reported to contain FIF. Most commonly, it presents with a single parasitic fetus as in our case; however, multiple fetuses ranging from 2 to 5 have also been reported [6–11].

Symptoms relate to the mass effect. Organs of different systems can be found in these fetuses. Commonly noticed organs are the vertebral column and limbs. However, other organs such as the ribs, central nervous systems, gastrointestinal tract, vessels, and occasionally thymic tissues can also be seen. The blood supply of the FIF is derived most commonly from the abdominal wall plexus, as the mass is attached to the abdominal wall. The size and weight of FIF varies depending on the blood supply. However, the absence of an independent circulatory system could account for fetal growth retardation in almost all cases. Brain tissue and intestines were detected in half the cases. Other uncommon organs reported are thyroid, parathyroid, pancreas, spleen, kidney, adrenal, testis, ovaries, urinary bladder, tongue, salivary glands, lymph nodes, trachea, and teeth [11].

In our case, the FIF was located in the upper retroperitoneum and weighing ~1500 grams. The sac was closely adhered to the undersurface of the liver, right kidney, and inferior vena cava. Similarly, microscopy of FIF in our case consists of respiratory, intestinal epithelium, muscle bundles, cartilages, bones, glial tissue, adipocytes, thyroid, and lymphoid follicles.

Fetus in fetu and well-formed teratoma having all three germ layer components are a matter of dispute for their independent existence. “Willis criteria” explain the differences between the two, on the basis of an axial skeleton with vertebral axis development (explaining embryological development passing through the stage of primitive streak) and an appropriate arrangement of other organs and limbs with respect to the axis in FIF [5, 12]. To be called fetus in fetu, the mass must demonstrate true organogenesis. Nonvisualization of the vertebral axis on radiography or CT scan does not exclude fetus in fetu as the pathologist can see it. Fetus in fetu masses show varying degrees of organ system differentiation and deformity [13].

Molecular karyotyping and genetic analysis of such fetiform mass also help in the diagnosis of fetus in fetu. However, in this case, it could not be done due to its unavailability in the country. Microsatellite markers, single nucleotide polymorphism (SNP), differentially methylated region (DMR) within the human IGF2-H19 locus analysis, and polymerase chain reaction (PCR) are few of the methods for genetic analysis in FIF to further support the diagnosis and confirm its monozygotic twin origin. These analyses help to establish the same genotype between the host and its fetiform mass as they both inherit one copy each of alleles from the parent. This will also be helpful to distinguish FIF from teratoma as the genetic analysis of teratoma could show sequences different from the host as most of them are derived from totipotent germ cells after the first meiotic division [14].

FIF should also be differentiated from ectopic pregnancy in adults. Finding of an elevated *β*-human chorionic gonadotrophin level and the documentation of chorionic tissue can further differentiate FIF from ectopic pregnancy. In our case, *β*-HCG level was normal with no evidence of chorionic tissue on histopathology.

Cross-sectional imaging has a valuable role in diagnosing FIF. Nocera et al. [13] described the CT findings of FIF for the first time in their study. CT could easily identify FIF as a well-defined mass with central bony structures surrounded by round or tubular collection of fat. The presence of vertebrae or long bones is necessary for the provisional diagnosis of FIF. CT is also helpful in the identification of the mass effects and structural relationship of FIF with the surrounding structures. Study of bony structures in FIF can be precisely done by three-dimensional CT with volume rendering reconstructions [15].

## 4. Conclusions

The case presented in our report meets all the accepted criteria of an abdominal FIF. The preoperative diagnosis of FIF is based on the observation of limbs or vertebral column in a mass on imaging modalities. The treatment of choice for FIF is complete resection with follow-up in certain cases. Further researches and workup including cytogenetic analysis need to be done to demonstrate the true characterization of FIF. Further studies to determine the possible association between FIF and highly differentiated teratoma are also needed.

## Data Availability

The data used to support the findings of this study are available from the corresponding author upon request.
